# Nucleocytoplasmic Shuttling of Histone Deacetylase 9 Controls Activity-Dependent Thalamocortical Axon Branching

**DOI:** 10.1038/s41598-017-06243-7

**Published:** 2017-07-20

**Authors:** Ricardo Alchini, Haruka Sato, Naoyuki Matsumoto, Tomomi Shimogori, Noriyuki Sugo, Nobuhiko Yamamoto

**Affiliations:** 1 Neuroscience Laboratories, Graduate School of Frontier Biosciences, Osaka University, 1-3 Yamadaoka, Suita, Osaka 565-0871 Japan; 2 Riken Brain Science Institute, Wako, Saitama 351-0198 Japan; 3 Montreal Neurological Institute, McGill University, Montreal, Quebec H3A 2B4 Canada

## Abstract

During development, thalamocortical (TC) axons form branches in an activity-dependent fashion. Here we investigated how neuronal activity is converted to molecular signals, focusing on an epigenetic mechanism involving histone deacetylases (HDACs). Immunohistochemistry demonstrated that HDAC9 was translocated from the nucleus to the cytoplasm of thalamic cells during the first postnatal week in rats. In organotypic co-cultures of the thalamus and cortex, fluorescent protein-tagged HDAC9 also exhibited nuclueocytoplasmic translocation in thalamic cells during culturing, which was reversed by tetrodotoxin treatment. Transfection with a mutant HDAC9 that interferes with the translocation markedly decreased TC axon branching in the culture. Similarly, TC axon branching was significantly decreased by the mutant HDAC9 gene transfer *in vivo*. However, axonal branching was restored by disrupting the interaction between HDAC9 and myocyte-specific enhancer factor 2 (MEF2). Taken together, the present results demonstrate that the nucleocytoplasmic translocation of HDAC9 plays a critical role in activity-dependent TC axon branching by affecting transcriptional regulation and downstream signaling pathways.

## Introduction

During development, neurons initially project their axons following a genetically defined developmental program, while neural activity refines neuronal circuits at later developmental stages. Axon branching is a phenomenon which represents the refinement process in the developing brain. The thalamocortical (TC) projection is one of the best-characterized systems of activity-dependent axon branching, exemplified by eye-specific projections in the primary visual cortex of certain higher mammals and barrel-specific projections in the somatosensory cortex of rodents^1–8^.

Our previous studies have shown that neuronal activity such as evoked and spontaneous activity acts as a positive regulator for TC axon branching^9, 10^. Moreover, manipulation of spontaneous firing activity has demonstrated that both presynaptic (thalamic cells) and postsynaptic synaptic cell (cortical cells) activity is necessary^11, 12^. As for the molecular mechanism, postsynaptic cell activity has been shown to release branch-promoting molecules from these cells themselves^13^. However, the presynaptic mechanism by which neural activity modifies axonal branching remains largely unknown, although several factors including cytoskeleton-regulatory molecules have been shown to contribute to branch emergence^14, 15^.

An intriguing view of activity-dependent axon branching is transcriptional regulation by neuronal activity^16^. The class IIa histone deacetylases (HDACs) attracted our attention as tentative mediators of neural activity in axonal remodeling because of their role in the interplay between neural activity and gene expression^17^. While HDACs primarily act in the nucleus by modifying histones, which results in suppression of gene expression, class IIa HDACs (HDAC4, 5, 7 and 9) form a distinct phylogenetic group, characterized by their ability to shuttle between the nucleus and the cytoplasm^18, 19^. This nucleocytoplasmic translocation is evoked by calcium influx, such as that downstream of neuronal activity, resulting in phosphorylation of the amino-terminal extensions of HDACs and leading to their association with 14-3-3 adaptor proteins and the nuclear export of protein complexes containing HDACs^20, 21^. Class IIa HDACs’ activity-dependent gene regulation is attributed to their inhibitory effects on myocyte-specific enhancer factor (MEF)-2-dependent transcription^22^. Notably, HDAC9 is abundantly expressed in the brain^23^. Its predominant isoform lacks the deacetylase catalytic domain and acts solely by recruiting other HDACs and subsequently repressing MEF2-mediated gene expression^24, 25^. Our previous work has further demonstrated that activity-dependent HDAC9 nucleocytoplasmic translocation contributes to dendritic growth in cortical neurons^26^, suggesting that a similar mechanism works on axon branching.

In the present study, we investigated the possibility that HDAC9 mediates presynaptic activity of thalamocortical axon branch formation by examining the endogenous expression of HDAC9 and manipulating its translocation using mutants. First, HDAC9 expression and subcellular localization were examined in the developing thalamic nuclei. Second, activity-dependent translocation between the nucleus and cytoplasm and its involvement in axon branching were investigated in *in vitro* preparations using HDAC9 mutants. Finally, we show evidence that HDAC9 translocation contributes to TC axon branching *in vivo*.

## Results

### HDAC9 translocation between the nucleus and cytoplasm

**Figure 1:**
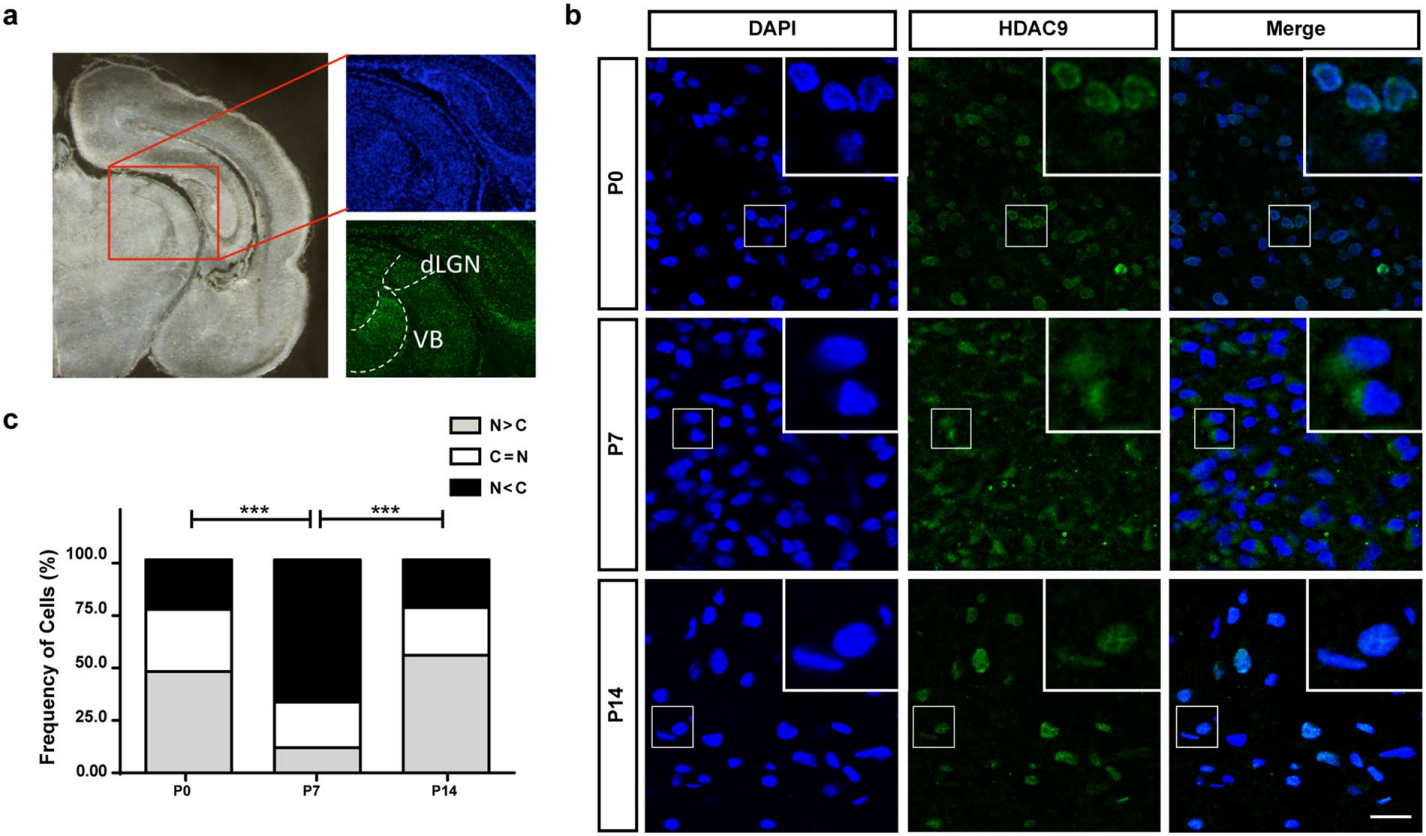
HDAC9 translocation in the developing rat thalamus. (**a**) Locations of dLGN and VB are shown in a coronal section. (**b**) P0, P7 and P14 rat thalami were analyzed after immunohistochemistry against HDAC9. HDAC9 export from the nucleus can be observed by the end of the first postnatal week. Scale bar: 35 μm Green: HDAC9. Blue: DAPI. (**c**) Distribution of the cells according to the ratio of HDAC9 signal intensity in the cytoplasm normalized to its nuclear intensity. Cells in which the cytoplasm/nuclear signal ratio was below 0.9 were considered as having a nuclear signal (N > C). Cells that the average signal in the nucleus and the cytoplasm did not differ in more than 10% (i.e. ratios between 0.9 and 1.1) were categorize as N = C. Cells in which the signals in the cytoplasm were considerably higher than in the nucleus (ratios above 1.1) were categorized as N < C. N = 958, 2029 and 808 cells from 3 different animals for P0, P7 and P14, respectively. Statistical analysis by overall Chi-square (Chi = 898.7, with 4 degrees of freedom, p < 0.0001) followed by pairwise Chi-square with Bonferroni correction. ***p < 0.001.

Based on previous reports of mRNA expression of *Hdac9* in the brain^23, 24^, we examined its protein expression in two developing sensory thalamic nuclei, the dorsal lateral geniculate nucleus (dLGN) and the ventrobasal complex of the thalamus (VB) (Fig. 1a). For this, coronal sections were subjected to immunohistochemistry with anti-HDAC9 and counter-stained with DAPI. As shown in Fig. 1b, the signal was mostly cytoplasmic at postnatal day (P) 7, whereas it was more distributed in the nucleus at P0. Based on the subcellular distribution, thalamic cells were classified into three different categories: predominantly nuclear (N > C), nearly equally distributed between nucleus and cytoplasm (N = C), and predominantly cytoplasmic (N < C) (Fig. 1c). The cytoplasmic distribution (N < C) was found only in less than one-quarter (23.3%, n = 958 cells from 3 animals) of the cells at P0, but was in the majority (66.8%, n = 2029 cells) at P7. This indicates that HDAC9 translocates from the cell nucleus to cytoplasm during the first postnatal week. However, HDAC9 signals in the cytoplasm were reduced in thalamic nuclei at P14 (22.5%, n = 808 cells), when HDAC9 was again distributed predominantly in the nucleus.

**Figure 2:**
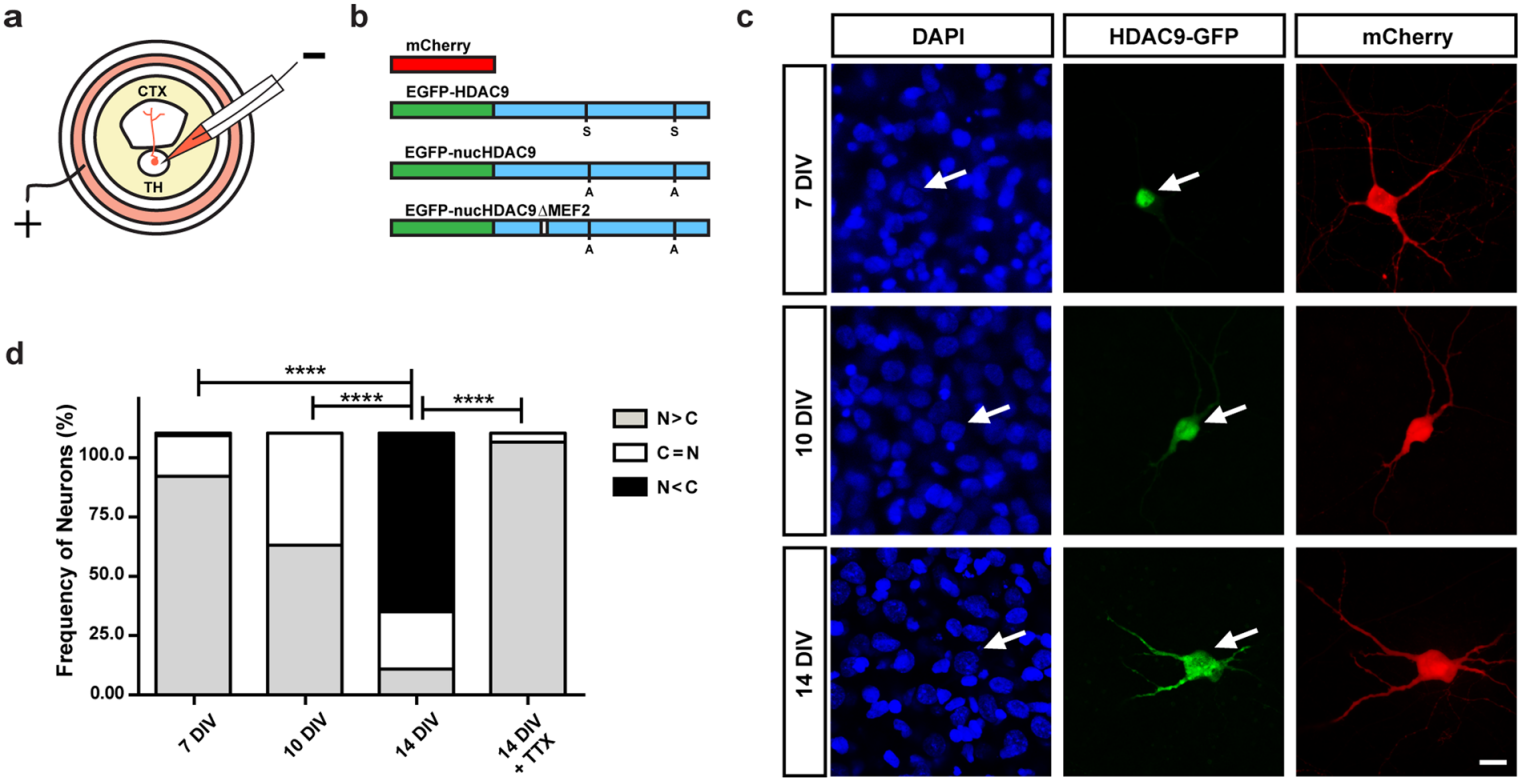
Subcellular localization of HDAC9-EGFP fusion protein in TC organotypic co-culture preparations. (**a**) Experimental set-up of the co-culture preparation and electroporation into thalamic cells. (**b**) Schematic representation of the plasmids used in the present study (see the methods). (**c**) Representative thalamic neurons at 7, 10 and 14 DIV. HDAC9-EGFP distribution in the cells can be compared to the cytoplasmic protein mCherry and the nuclear staining of DAPI. Arrows refer to the position of the same cell at a given time point. Scale bar 10 μm. (**d**) Quantification of subcellular localization of HDAC9 was expressed as the ratio of transfected cells to illustrate HDAC9-EGFP signal concentrated predominantly in the nucleus (N > C), equally in the nucleus and the cytoplasm (N = C) or predominantly in the cytoplasm (N < C). Statistical analysis by overall Chi-square (Chi = 158, with 6 degrees of freedom, p < 0.000001) followed by pairwise Chi-square with Bonferroni correction. ****p < 0.0001.

To reveal the mechanisms underlying HDAC9 nucleocytoplasmic translocation, we studied the subcellular localization of HDAC9 during development in cultured thalamic neurons. To do this, we prepared organotypic co-cultures of the thalamus and cortex (Fig. 2a), which recapitulate cellular organization and development *in vivo* ^10, 27^. HDAC9-EGFP and mCherry plasmids were co-transfected into thalamic neurons via local electroporation (Fig. 2a,b). After 7–14 days in culture (corresponding to P0-7 *in vivo* for thalamic cells), the HDAC9-EGFP signal was observed in the nuclear and cytoplasmic regions of thalamic neurons (Fig. 2c). Based on the subcellular distribution of HDAC9-EGFP, transfected thalamic cells were classified into three different categories, as described above (Fig. 2c,d). At 7 days *in vitro* (DIV), HDAC9-EGFP was predominantly distributed in the nucleus of most thalamic cells tested (83.5%, n = 100 cells from 3 cultures). At 10 DIV, HDAC9-EGFP was almost equally distributed between the nucleus and cytoplasm in nearly half of cells (42.8%, n = 30 cells from 3 cultures), although nuclear localization was predominant in the remaining fraction. At 14 DIV, the majority (68.3%, n = 85 cells from 5 cultures) displayed stronger HDAC9-EGFP signal in the cytoplasm than in the nucleus. In these cells, HDAC9-EGFP was found to spread into most of the neurites (Fig. 2c). Thus, the nucleocytoplasmic translocation of HDAC9 in cultured thalamic neurons is consistent with that *in vivo*.

We previously demonstrated that neuronal activity induces nucleocytoplasmic HDAC9 translocation in cortical neurons, and that blocking this activity drives HDAC9 back to the nucleus^26^. To determine whether the nucleocytoplasmic shuttling in thalamic neurons is dependent on spontaneous firing, whose frequency increases during the second week in culture^10^, the sodium channel blocker, tetrodotoxin (TTX) was added to the culture medium at 13 DIV, and the subcellular localization of HDAC9 was examined on the next day. While HDAC9 was primarily distributed in the cytoplasm at 14 DIV, TTX addition caused HDAC9 to be confined to the nucleus in virtually all cells tested (96.6%, n = 57 cells from 3 cultures) (Fig. 2d, Suppl. Figure 1). Therefore, TTX treatment restored HDAC9 distribution to the nucleus, indicating that neuronal activity regulates HDAC9 shuttling reversibly between the nucleus and cytoplasm of thalamic neurons.

### Influence of HDAC9 subcellular localization on axon branching

**Figure 3:**
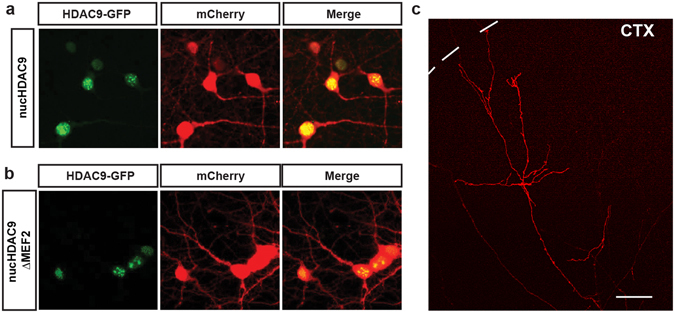
Subcellular localization of HDAC9 mutants in thalamic cells and TC axon branching *in vitro*. Both of nucHDAC9-EGFP (**a**) and nucHDAC9ΔMEF2-EGFP (**b**) were distributed in the nuclei of thalamic cells at 14 DIV, while co-expressed mCherry was distributed in cell bodies and dendrites. Green: EGFP signal from the fusion protein. Red: mCherry. Scale bar, 5 μm. (**c**) Representative mCherry-labeled axon in the cortical explant, which elongated from the transfected thalamic cell. Interrupted line indicates the pial surface of the cortical explant. Scale bar 100 μm.

We investigated the role of HDAC9 translocation in TC axon branching in the co-culture preparations (Fig. 2a). Our previous studies have demonstrated that TC axons mostly form branches during the second week in the culture. Since HDAC9 translocation also takes place during the same developmental stage, we asked whether the relationship between HDAC9 translocation and axon branching is simply correlated or is causal. To address this issue, we used a dominant-negative form of HDAC9 which is unable to translocate from the nucleus to the cytoplasm due to two serine-to-alanine mutations at residues 218 and 448 (Fig. 2b). This substitution impairs the phosphorylation of HDAC9 that is associated with nucleocytoplasmic translocation by its effector proteins, such as CaMK^21, 26^. Hence, even with the high-frequency neuronal activity that is normally observed in our culture system during the second week, the mutant HDAC9 should remain localized in the nucleus. This mutant protein, called nucHDAC9 (for nuclear, non-translocating HDAC9), was co-transfected with mCherry into a small number of thalamic cells. After confirming that nucHDAC9 was localized in the nucleus after 14 days in culture (Fig. 3a), we investigated branch formation of the axons that penetrated into the cortical slice (Fig. 3c).

**Figure 4:**
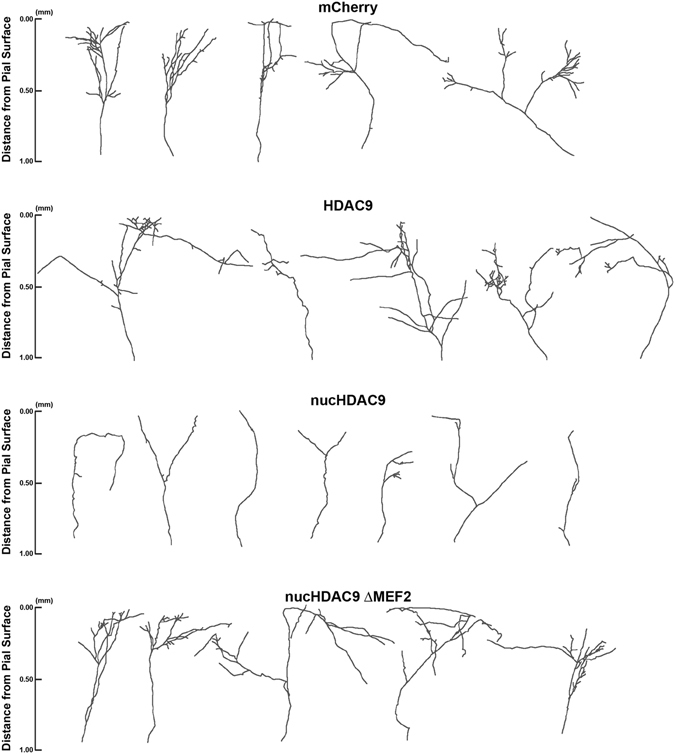
Disruption of HDAC9 translocation and MEF2 interaction affect TC axon branching. Representative tracings of control and mutant HDAC9 transfected axons. Thalamic cells were electroporated with only mCherry to observe control TC axon branching. To examine the effect of the HDAC9 mutants, mCherry was co-transfected with either of HDAC9, nucHDAC9, or nucHDAC9ΔMEF2.

**Figure 5:**
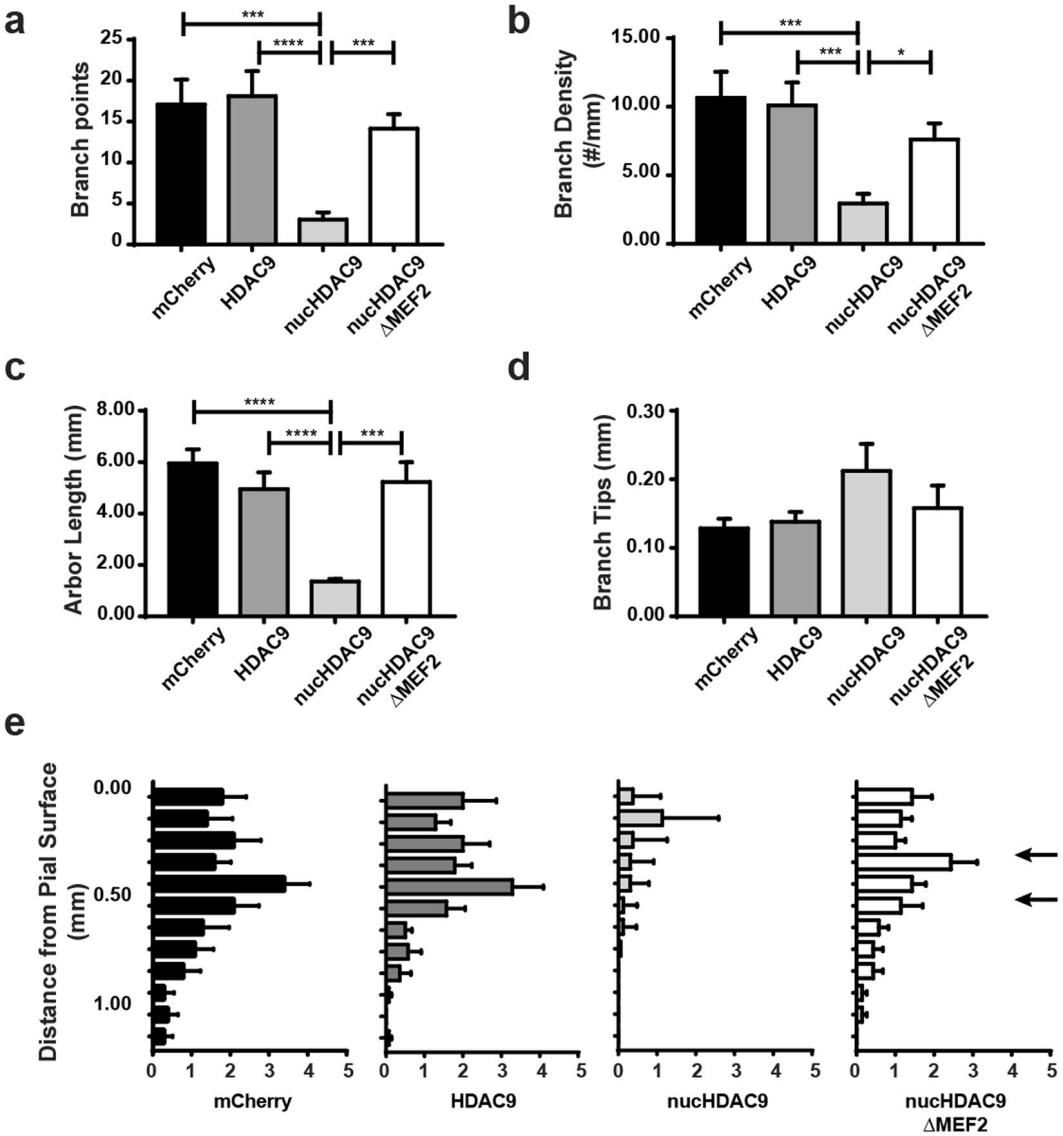
Nucleocytoplasmic translocation of HDAC9 is required for TC axon branching via MEF2 regulation. (**a**) Average number of branch points per axon. Thalamic cells were electroporated with only mCherry, HDAC9 co-electroporated with mCherry, nucHDAC9 co-electroporated with mCherry, and nucHDAC9ΔMEF2 co-electroporated with mCherry. N = 12, 17, 18 and 14 axons from 4–6 co-cultures for each condition, respectively. (**b**) Branch density per axon. (**c**) Total branch length per axon. (**d**) Branch tip length. Statistical analysis by Kruskal-Wallis test with Dunn correction comparing the 4 columns. *p < 0.05. ***p < 0.001. ****p < 0.0001. e. Laminar distribution of branching points according to their distance from the pial surface. Arrows indicate presumed layer 4. Statistical analysis by two-way ANOVA for whole column effects with Tukey’s multiple comparisons test confirmed a significant difference in laminar specificity between nucHDAC9 and control (p < 0.0001), while no significant difference was found between nucHDAC9ΔMEF2 and control or wild-type HDAC9.

Compared to cells transfected with only mCherry (control), those also transfected with nucHDAC9 displayed a drastic change in axon morphology (Fig. 4). Thalamic axons transfected with nucHDAC9 had significantly fewer branches (4.1 ± 0.9, n = 18 axons) than the control axons (17.8 ± 3.1, n = 12 axons) or axons from cells expressing wild-type HDAC9 (18.1 ± 3.0, n = 17 axons) (Figs 4 and 5a). Correspondingly, branch density (control: 10.7 ± 1.9 branches/mm, HDAC9: 10.1 ± 1.7 branches/mm, nucHDAC9: 2.9 ± 0.7 branches/mm) and the total branch length (control: 5.9 ± 0.5 mm/axon, HDAC9: 4.9 ± 0.6 mm/axon, nucHDAC9: 1.4 ± 0.1 mm/axon) were dramatically decreased in nucHDAC9-expressing thalamic axons (Fig. 5b,c). In contrast, tip length was not significantly changed (control: 0.128 ± 0.014 mm, HDAC9: 0.138 ± 0.014 mm, nucHDAC9: 0.212 ± 0.039) (Fig. 5d). This strongly suggests that HDAC9 translocation promotes TC axon branching.

### Requirement of HDAC9 interacting partners for axon branching

As HDAC9 was discovered as a repressor for MEF2, we hypothesized that the HDAC9 effect on axon branching is mediated by MEF2^25^. To address this, we studied TC axon branching by further removing the MEF2-binding domain, located in the N-terminal region of nucHDAC9 (Fig. 2b). This mutant protein, called nucHDAC9ΔMEF2 (or non-translocating, non-MEF2-binding HDAC9), was co-transfected with mCherry into thalamic neurons. Subcellular localization and TC axon morphology were then analyzed as in the previous experiment.

Nuclear localization of nucHDAC9ΔMEF2 was also observed at 14 DIV in the transfected thalamic cells (Fig. 3b). Both constructs (nucHDAC9 and nucHDAC9ΔMEF2) showed similar expression levels, but axon branching was not severely affected by nucHDAC9ΔMEF2 transfection (Figs 4 and 5). Indeed, the axons transfected with nucHDAC9ΔMEF2 formed extensive branches (15.4 ± 1.8, n = 14 axons) (Fig. 5a), whose number was not significantly different from that in the mCherry control. Consistent with this result, nucHDAC9ΔMEF2 axons also displayed branch density, axon arbor length and branch tip length similar to the control axons (Fig. 5b–d).

Laminar specificity of axon branching was further investigated for TC cells expressing the HDAC9 mutants (Fig. 5e). In the control (mCherry only) and nucHDAC9ΔMEF2-expressing TC axons, branch points were distributed mostly in the upper cortical layers, peaking within 0.3–0.5 mm from the pial surface, which corresponds to the presumed layer 4^27^. In contrast, nucHDAC9 transfected axons formed branches in a distorted pattern (Fig. 5e), with homogeneously poor branching throughout all cortical layers. Statistical analysis confirmed the significant difference in laminar specificity between nucHDAC9 and the control, while no significant difference was found between nucHDAC9ΔMEF2 and control or wild-type HDAC9.

These results indicate that binding of HDAC9 to MEF2 is required for its inhibitory effect on axon branching.

### HDAC9 action on TC axon branching *in vivo*

**Figure 6:**
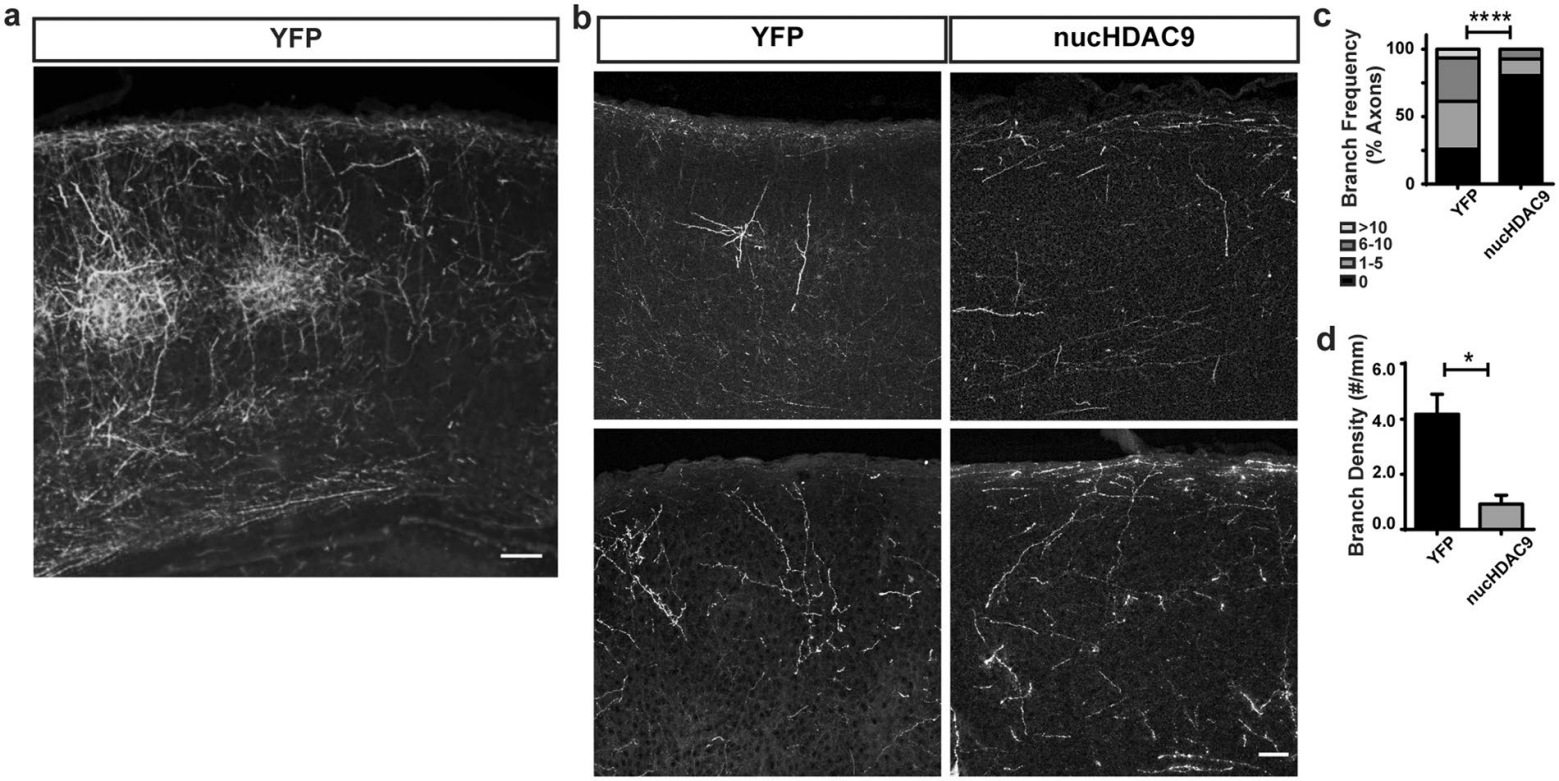
HDAC9 nuclear export enhances axon branching *in vivo*. (**a**) EYFP-labeled TC axonal projection in the P7 SSp cortex of an animal whose thalamus was electroporated with EYFP plasmid. Coronal section of approximately 60 μm thickness. Scale bar 100 μm. (**b**) Representative TC axon fragments from control (EYFP-electroporated) and nucHDAC9/mCherry co-electroporated neurons. Coronal sections of 20 μm thickness. Scale bar 50 μm. Statistical analysis by Chi-square (Chi = 22.3, with 3 degrees of freedom), p < 0.0001. (**c**) Distribution of axon segments according to their branch number. (**d**) Average branch density. Statistical analysis by Student *t*-test. *p < 0.05.

Finally, we examined whether HDAC9 translocation regulates TC axon branching *in vivo* by suppressing HDAC9 translocation in thalamic neurons. To do this, nucHDAC9 and mCherry were co-transfected into the dorsal thalamus, primarily into the VB in embryonic mouse brains^28^. The EYFP plasmid was similarly transfected into the thalamic regions as a control. These brains were dissected by the end of the first postnatal week, which corresponds to the period immediately after HDAC9 translocation. Labeled axonal processes were observed in brain sections (Fig. 6a,b).

In order to compare to our *in vitro* data, we focused on the projections to the primary somatosensory cortex (SSp), the target region for VB axons (Fig. 6a). Histological sections showed that abundant branches were formed along the barrel structures (Fig. 6a)^29^. Thinner histological sections (20 µm) containing sparsely labeled axons allowed us to assess branch formation by tracing axonal segments (Fig. 6b). Some branches, and occasionally extensive branches, were found in the control labeled axons (Fig. 6b,c). In contrast, no branches were observed in 80.5% of the axonal segments from nucHDAC9-transfected brains, and none of the fragments had more than 10 branches (Fig. 6b,c). Quantification of axonal branching showed that while control axons exhibited an average of 4.2 branches/mm in axonal fragments (n = 31 axons from 3 animals), this number decreased significantly to 0.9 branches/mm in mutant axons (n = 41 axons from 3 animals) (Fig. 6d). The orientations of axonal fragments were examined (see method), to confirm the absence of a sampling bias. In control two-thirds (21/31, 67.7%) of axonal fragments had a vertical orientation (−45 to 45 degrees), and a similar fraction of nucHDAC9 fragments (26/41, 63.4%) were vertically orineted. Therefore, it is unlikely that a sampling bias occurred.

Thus, TC axons also formed less branches *in vivo* by suppressing nucleocytoplasmic translocation of HDAC9.

## Discussion

**Figure 7:**
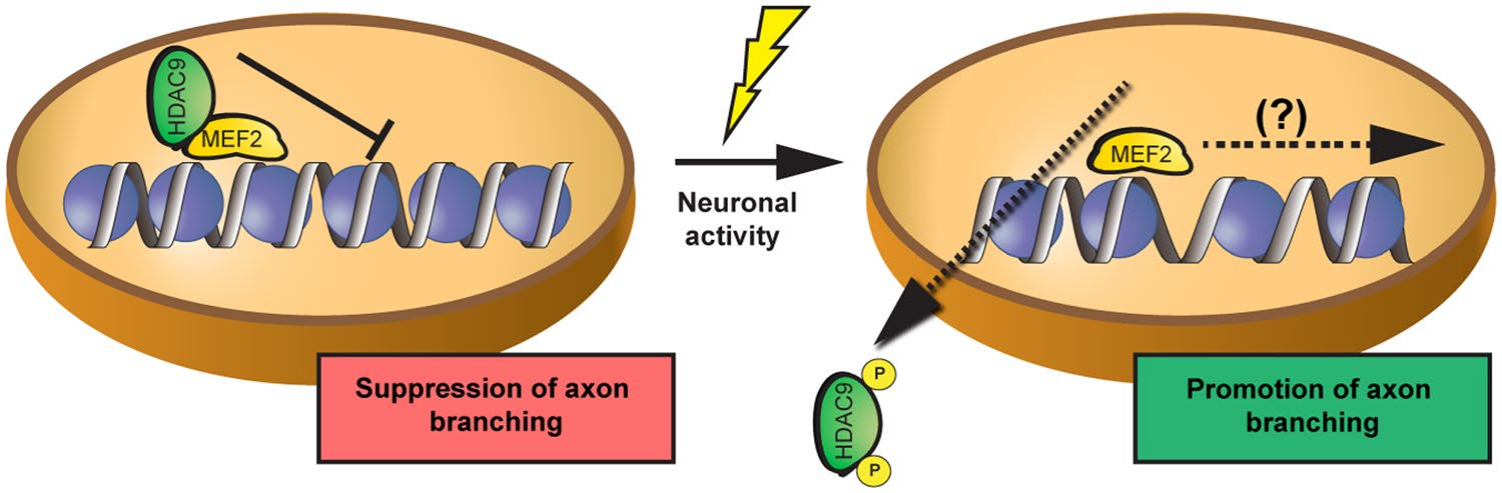
Working model of HDAC9 function in axon branch formation. HDAC9 is confined to the nucleus, where it can bind to transcription factors such as MEF2 and represses axon branching. Neuronal activity induces HDAC9 export to the cytoplasm, which promotes axon branching.

Altogether, our data suggest a pivotal role for HDAC9 nucleocytoplasmic translocation as a mechanism that converts neuronal activity into the remodeling of axons. In our model, neuronal activity results in the nucleocytoplasmic translocation of HDAC9, which hence promotes axon branching via interactions with transcription factors (Fig. 7).

Despite the high levels of spontaneous neuronal firing exhibited by organotypic preparations^10, 11^, a simple mutation of HDAC9 was enough to phenocopy a previous work in which neuronal activity was blocked, suggesting that HDAC9 is located downstream of it. Although it is unknown what neuronal activity *in vivo* is involved in TC axon branching, synchronous activity may be more efficient for HDAC9 translocation, as wave-like network activity is prominent at early postnatal stages^30–32^. Moreover, the reduction of HDAC9 signals in the cytoplasmic region at later developmental stages may be due to the alteration from the synchronous large activity to sparsification of firing^32^. Alternatively, this may be attributable to the nuclear localization of HDAC9 in later stages being dependent on its export by 14-3-3 proteins^20^. The developmental increase in phosphorylation levels of 14-3-3 s may antagonize their function in removing class IIa HDACs from the nucleus^33^. Therefore, further studies might help to unveil whether the presence of nuclear HDAC9 at P14 relies on electrophysiological or molecular properties of the thalamus.

While both pre- and postsynaptic activities participate in the remodeling of cortical circuitry including the TC projection^11, 34, 35^, much of what is known about the regulation of TC branching is restricted to the secretion of several molecules by the postsynaptic portion of this system^13, 36, 37^. Our work provides insights into how the pre-synaptic cell is also remodeled by neural activity through changes in its intrinsic factors.

Similarly, much of what we know about presynaptic effects of neuronal activity concerns the regulation of the actin cytoskeleton and neurotrophin expression^14, 15, 38^. As our previous work has demonstrated, HDAC9 regulates the expression of immediate early genes, such as c-fos, implicating them in the regulation of activity-dependent genetic programs, which could underlie the effects that we observed. HDAC9 has also been implicated in the regulation of activity-dependent genes in a genome-wide screen for activity-dependent enhancers based on the regulation of FOS^39^.

Broad-spectrum HDAC inhibition has been shown to impair central nervous system axon outgrowth and regeneration^40, 41^, suggesting that some HDACs are detrimental to axonal development. Our results relate HDACs to a restrictive function in activity-dependent axon branching, while also adding HDAC9 to the list of HDACs that may developmentally impair CNS axonal growth and regrowth after damage.

Notably, another class IIa HDAC, HDAC5, translocates to the cytoplasm upon axotomy, to regulate peripheral nerve axonal regeneration^42, 43^. In that system, damage to the axonal membrane results in a calcium signaling cascade similar to that which regulates HDAC9. In this context, HDAC effects on cytoskeleton deacetylation have been shown to be positive on axonal outgrowth and regrowth, while confinement of HDACs to the nucleus likewise inhibited axonal sprouting^42–44^. Due to the presence of endogenous HDAC9 translocation in our electroporated neurons, we believe that the transcriptional, nuclear role of HDAC9 on axon branching surpasses its possible effects in the cytoplasm. The lack of a deacetylase domain in the most abundant isoform of HDAC9 indicates that its key function is to serve as a recruiter of other transcriptional regulators, such as MEF2, HDAC3, Sin3, Ikaros and CoREST, while its inferred role of regulating chromatin structure may be an effect of the complex of these factors^25, 45–49^. Interestingly, HDAC3 has been related to the auditory critical period, and the confinement of HDAC9 to the cell nucleus at later stages of development in the dLGN might raise the possibility that such HDAC9 complex could play a similar role in the establishment of the visual critical period^50^.

In our model, the type of interaction between HDAC9 and transcription factors seem to play a much more decisive role in the regulation of axon branching than the expression levels of such protein. While HDAC9 overexpression did not cause any axon branching phenotype, nucHDAC9 significantly inhibits axon branches formation, and the effect was rescued by removing MEF2 interaction site. Among the transcription factors modulated by HDAC9, MEF2 seems to be the most prominent, as HDAC9 itself was initially identified as MITR, an acronym for MEF2-interacting transcriptional repressor^23, 25, 51^. Based on the well-known property we studied the effect of the interaction on axon branching. Interestingly, removing the ability of HDAC9 to bind to MEF2 greatly rescued its detrimental effects on axon branching, suggesting the mandatory participation of this transcription factor downstream of HDAC9. The effects of MEF2 on neural activity-dependent gene expression are widely known^52–54^. Interestingly, most reports of MEF2 acting on neuronal development have linked its expression to the restriction of synapse formation. In contrast, our data may indicate that the MEF2 family of transcription factors promotes the formation of activity-dependent axon branches. MEF2 has been suggested to act as a metaplasticity sensor, in which changes in the pattern of neural activity are more indicative of its role in gene regulation than the presence of activity itself, which can explain the variable in MEF2 roles in different systems^53^. Our tentative model may be more relevant to the early stages of thalamic development, and is reminiscent of the function of the MEF2/HDAC9 interaction during muscular differentiation, in which electrical activity induces HDAC9 translocation, thus enhancing the maturation of skeletal muscle cells through MEF2-regulated gene expression^17, 55^. As described above, HDAC9 also interacts with other components, which could affect downstream functions.

Thus, further studies on the regulatory role of HDAC9-containing complexes during thalamic development should help to elucidate which genes and proteins are regulated by neural activity during the formation of TC projections.

## Methods

### Animals

C57BL/6Cr mice and Sprague–Dawley rats were used (NIHON CLEA, Tokyo, Japan). Noon of the day on which the vaginal plug was detected in the morning was designated embryonic day 0 (E0). The first 24 h after birth was referred to as postnatal day 0 (P0). All experiments were performed according to guidelines laid down by animal welfare committees of Osaka University and the Japanese Neuroscience Society. The protocol was approved by the Committee on the Ethics of Animal Experiments of Osaka University (Permit Number: FBS 07–037).

### Immunostaining

Immunostaining was performed to enhance the fluorescence of electroporated neurons in organotypic cultures or brain sections. Cultures were fixed with 4% PFA in 0.1 M phosphate buffer containing 0.15 M NaCl (PBS) for 2–3 h at room temperature (RT), washed five times in PBS, and then incubated in PBS containing 5% normal goat serum and 0.3% Triton X-100 for 1 h at RT. Cultured slices on a membrane were cut out from the insertion and incubated with monoclonal rat anti-GFP IgG2a (1:1500, Nacalai Tesque) or rabbit anti-RFP polyclonal antibody (1:1500, MBL International) in blocking buffer overnight at 4 °C. After five washes in PBS containing 0.3% Triton X-100, the cultures were incubated with Alexa Fluor 488-conjugated goat anti-rat IgG (1:500; Invitrogen) or Cy3-conjugated donkey anti-rabbit IgG (1:400, Chemicon) for 2 h at RT. After five washes, the slices were mounted on glass slides and encapsulated with coverslips.

For *in vivo* analysis of HDAC9 nucleocytoplasmic translocation, a similar immunohistochemistry protocol was carried out. After transcardial perfusion and post-fixation of the brain samples with 4% PFA in PBS for 2 h at 4 °C, 10-μm-thick coronal cryosections were prepared, blocked for 1 h at RT, and incubated with an anti-HDAC9 N-terminus polyclonal serum (1:50, Abgent) for 48 h at 4 °C. After being extensively washed, the sections were incubated with Alexa 488-conjugated anti-rabbit IgG (1:500, Invitrogen) for 24 h at 4 °C. Samples were then washed five times and mounted on glass slides.

### Quantification of HDAC9 subcellular localization

Quantification of HDAC9 subcellular localization *in vivo* was performed using the CellProfiler, following a previously published protocol^56^. In brief, nuclear staining was used to create masks for measuring the intensity of HDAC9 signal in each cell. HDAC9 fluorescence was measured inside the nuclear region (nuclear signal) or within a continuous region in the vicinity of the nucleus (cytoplasmic signal). CellProfiler calculated the ratio of cytoplasmic and nuclear signals for each cell and the results were plotted according the cells whose cytoplasmic/nuclear ratio was under 0.9 were considered as N > C, cells whose HDAC9 signal was nearly identical inside and outside the nucleus (ratios between 0.9 and 1.1) were considered as N = C, and cells whose cytoplasmic signals were clearly cytoplasmic (ratios superior to 1.1) were considered as N < C, following the method of categorization of HDAC9 localization that has been previously utilized in the literature^26, 57^.

For the experiments in which HDAC9-EGFP subcellular localization was observed *in vitro*, cells with a neuronal morphology were selected in the organotypic preparations and qualitatively scored following a previously described protocol^26, 57^. In brief, cells whose HDAC9 signal was concentrated in the nucleus are labelled as N > C, cells that had an even distribution of HDAC9 in the cell body are considered as N = C and cells whose HDAC9 signal was strongly spread throughout the cytoplasm (often including the neurites) were counted as N < C.

### Plasmids

The pCAG-EYFP, pCAGGS-HDAC9-EGFP and pCAGGS-HDAC9-S218/448A-EGFP constructs were previously described^9, 26^. To generate a vector expressing HDAC9 lacking the MEF2-binding domain, a deletion corresponding to amino acid residues 135–152 of HDAC9 cDNA was introduced into pCAGGS-HDAC9-S218/448A-EGFP by PCR using the mutagenic primer 5′-AGCTTCCTCCTCTCAGAGGCAAAGATAGAGGACGAAGTAAATCAGCAACAAAAGACACTCCAA-3′ and its antisense strand^25^.

The pCAG-mCherry plasmid was constructed by PCR amplification of the mCherry ORF from mCherry-C1 vector (Clontech) and ligation into a pGEM-T Easy Vector (Promega, Madison, WI), followed by subcloning of the ORF into the EcoRI site of the pCAGGS vector.

### Organotypic slice culture and gene delivery

TC organotypic co-cultures were prepared as described previously^27, 58^. In brief, the dorsal thalamic region was dissected from E15 rat embryos, and cortical slices were dissected from the visual and somatosensory cortices of P1 – P3 rats. A block of the thalamus was plated at the ventricular side of the cortical slice on a rat tail collagen-coated membrane filter (Millicell-CM PICMORG50; Millipore, Bedford, MA). The culture medium consisted of a 1:1 mixture of DMEM and Ham’s F-12 (Invitrogen, Carlsbad, CA) with several supplements containing insulin and transferrin^27^. These cultures were maintained at 37 °C in an environment of humidified 95% air and 5% CO_2_.

To label individual TC axons, plasmids of interest were transfected into a small number of thalamic cells using a previously described method, which makes it possible to observe individual axon morphology^9, 10^. In brief, at 2–3 days *in vitro* (DIV) the plasmid solution (1.0–4.5 μg/μl) was applied to the thalamic explants through a glass micropipette (tip diameter of 50 μm), and then electrical pulses (10 trains of 200 square pulses of 1 ms duration at 200 Hz, 400 μA) were delivered across a silver wire placed inside a glass micropipette (tip diameter of 100–200 μm) and a silver wire ground electrode placed in the culture medium. For the co-electroporation experiments, the HDAC9 construct of interest was added in a 3:1 ratio to mCherry, which resulted in a co-transfection rate in greater than 85% of cells. The electroporation was applied at 3 sites in each thalamic explant. mCherry-labeled axons were observed by confocal microscopy (D-ECLIPSE C1 confocal laser scanning microscope, Nikon) equipped with 10x and 40x objectives and the appropriate filters. Z-series of 2–30 optical sections were sampled.

### Axon branching quantification *in vitro*

Individually distinguishable axons that reached or passed through layer 4 were selected, and drawn using the NeuronJ plug-in for ImageJ^59^. We analyzed the following parameters: the number of branch points, the branch point density (number of branch points divided by the length of the axon shaft), the total length of the axons arbors and the branch tip length. Branch tip length refers to the distance from each axon tip to its closest branch point. The axons that did not branch were excluded from this analysis. The laminar locations of branch points were determined based on lower-magnification pictures. The cortical thickness was divided in 100 μm bins, and the number of branch points was counted in each bin.

### *In utero* electroporation and branch analysis *in vivo*

Thalamic electroporation followed a previously described method^28, 60^. In brief, the abdomens of timed-pregnant mice were opened, and ~E11 embryos were viewed with a fiber optic light source through the uterine wall. To examine the mutant HDAC9 activity, 1–2 µl of DNA (pCAGGS-HDAC9-S218/448A-EGFP at 2 µg/µl and pCAGGS-mCherry at 1 µg/µl) were injected into the third vesicle of each embryo and electroporated using 3 mm tweezer-type electrode electrodes (four square-wave current pulses, 30 V, 100 ms). For control, only pCAGGS-EYFP (1 µg/µl) was used for the transfection. The embryos were allowed to develop *in utero* and after birth. Brains were collected at P7 and fixed with 4% paraformaldehyde (PFA) for 3 h at 4 °C. Coronal cryostat sections of 20 μm or 60 μm thickness were prepared, and the tissue was then processed for immunohistochemistry as described. Axonal fragments were traced from sparsely labeled regions in the primary somatosensory cortex using ImageJ (see above), and the results were expressed as branch density (number of branches per length of labeled process). The axonal fragments which traveled horizontally within layer 1 and the white matter were excluded from the analysis. In addition, we split axon fragments according to the number of branches and plotted the results as the frequency at which a given number of branches occurred. The orientation of each axonal fragment was also examined, by defining fragments in which the main shaft orientation ranged from −45 to 45 degrees as “vertical”, and those ranging from −90 to −45 or 45 to 90 degrees as “horizontal”.

### Statistical analysis

Chi-square test was utilized to compare HDAC9 distribution *in vivo* and *in vitro* at different time points. For pairwise comparisons, Chi-square test was followed by Bonferroni correction in order to adjust the α level at 0.05^61^. Chi-square test and Student *t*-test were used for analysis of branch number *in vivo*. Kruskal-Wallis test with Dunn correction was utilized for *in vitro* analysis of branch number, density, total branch lengths and branch tip length. Two-way ANOVA with Tukey’s multiple comparisons test was used for analysis of laminar specificity. All statistical analysis was performed using Prism 7.0 (GraphPad).

## Electronic supplementary material

Supplementary Figure 1
