# Supplementary material for: Nucleocytoplasmic Shuttling of Histone Deacetylase 9 Controls Activity-Dependent Thalamocortical Axon Branching
Source: Sci Rep. 2017 Jul 20;7:6024. doi: 10.1038/s41598-017-06243-7 (PMC5519695; doi:10.1038/s41598-017-06243-7)
Supplement: Supplementary file 1 — Supplementary Figure 1 [file 41598_2017_6243_MOESM1_ESM.pdf]

Supplementary Information

Nucleocytoplasmic Shuttling of Histone Deacetylase 9 Controls Activity-Dependent Thalamocortical Axon Branching

Ricardo Alchini, Haruka Sato, Naoyuki Matsumoto, Tomomi Shimogori, Noriyuki Sugo, Nobuhiko Yamamoto

Supplementary Figure 1. Representative thalamic neurons at 14 DIV (control) or 14 DIV (TTX-treated). HDAC9-EGFP distribution in cells can be compared to nuclear staining with DAPI. Arrows refer to the position of the same cell at a given time point. Scale bar, 10 μm.

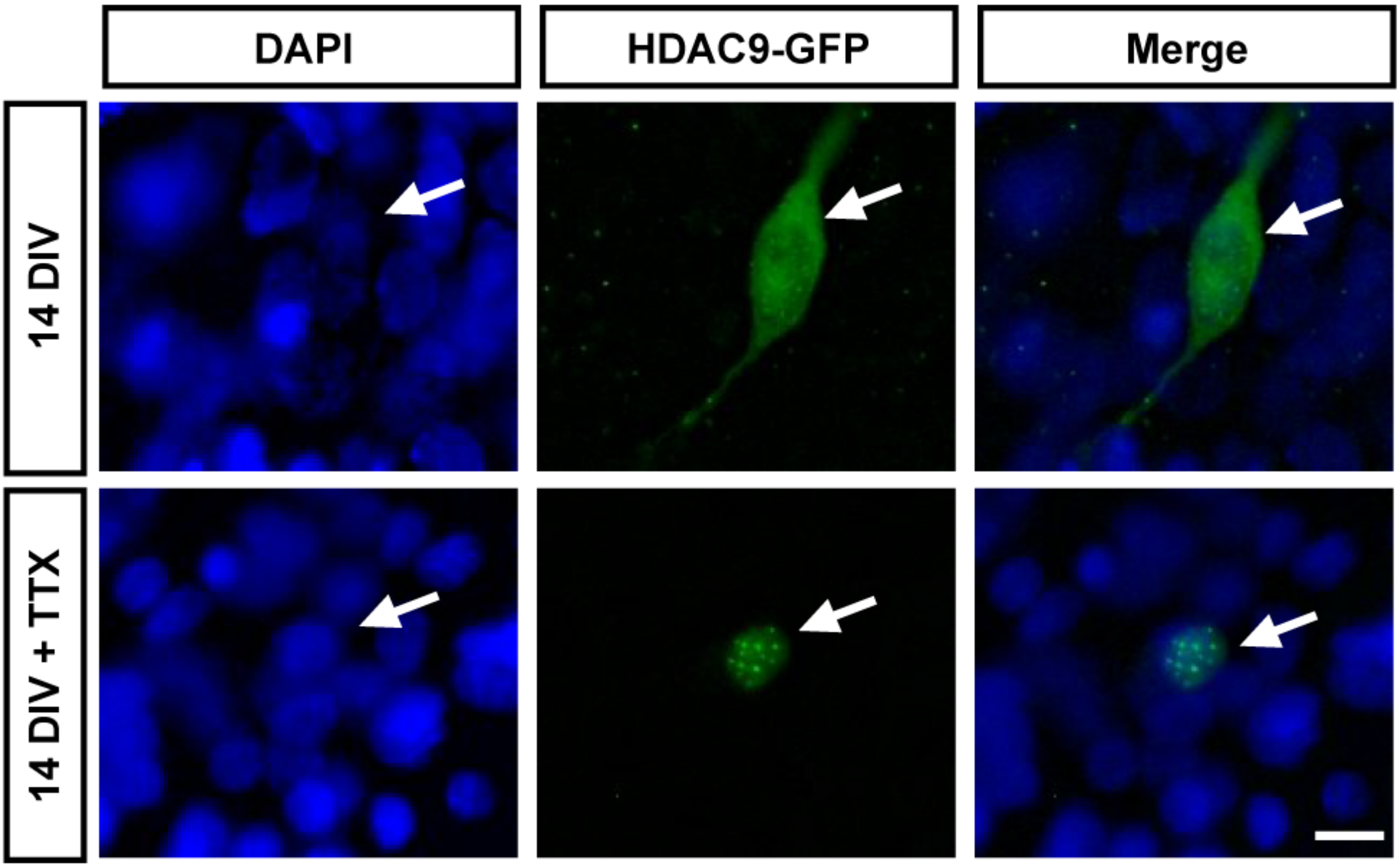

Supplementary Figure 1. Representative thalamic neurons at 14 DIV (control) or 14 DIV (TTX-treated). HDAC9-EGFP distribution in cells can be compared to nuclear staining with DAPI. Arrows refer to the position of the same cell at a given time point. Scale bar, 10 μm.
